# Molecular Characterization of Peroxidase (*PRX*) Gene Family in Cucumber

**DOI:** 10.3390/genes15101245

**Published:** 2024-09-25

**Authors:** Weirong Luo, Junjun Liu, Wenchen Xu, Shenshen Zhi, Xudong Wang, Yongdong Sun

**Affiliations:** 1 School of Horticulture and Landscape Architecture, Henan Institute of Science and Technology, Xinxiang 453003, China; 2 Henan Province Engineering Research Center of Horticultural Plant Resource Utilization and Germplasm Enhancement, Xinxiang 453003, China

**Keywords:** cucumber (*Cucumis sativus* L.), peroxidase (*PRX*), bioinformatics analysis, expression analysis

## Abstract

**Background**: The Peroxidase (*PRX*) gene family is essential for plant growth and significantly contributes to defense against stresses. However, information about *PRX* genes in cucumber (*Cucumis sativus* L.) remains limited. **Methods**: In this present study, *CsPRX* genes were identified and characterized using bioinformatics analysis. The expression pattern analysis of *CsPRX* genes were examined utilizing the RNA-seq data of cucumber from public databases and real-time quantitative PCR (qRT-PCR) analysis. **Results**: Here, we identified 60 *CsPRX* genes and mapped them onto seven chromosomes of cucumber. The CsPRX proteins exhibited the presence of 10 conserved motifs, with motif 8, motif 2, motif 5, and motif 3 consistently appearing across all 60 CsPRX protein sequences, indicating the conservation of CsPRX proteins. Furthermore, RNA-seq analysis revealed that differential expression of *CsPRX* genes in various tissues. Notably, a majority of the *CsPRX* genes exhibited significantly higher expression levels in the root compared to the other plant tissues, suggesting a potential specialization of these genes in root function. In addition, qRT-PCR analysis for four selected *CsPRX* genes under different stress conditions indicated that these selected *CsPRX* genes demonstrated diverse expression levels when subjected to NaCl, CdCl_2_, and PEG treatments, and the *CsPRX17* gene was significantly induced by NaCl, CdCl_2_, and PEG stresses, suggesting a vital role of the *CsPRX17* gene in response to environmental stresses. **Conclusions**: These findings will contribute valuable insights for future research into the functions and regulatory mechanisms associated with *CsPRX* genes in cucumber.

## 1. Introduction

Peroxidases (EC 1.11.1.X) belong to a significant class of isomerases that utilize hydrogen peroxide (H_2_O_2_) as an oxidizing agent to facilitate the oxidation of a variety of substrates. These enzymes are prevalent across a diverse range of living organisms, including plants, animals, and microorganisms. Peroxidases can be categorized into two primary types: hemoglobin peroxidases and non-hemoglobin peroxidases, based on their structural and catalytic properties [1]. Furthermore, the non-animal hemoglobin peroxidases can be divided into three categories: class I, class II, and class III [2]. Specifically, class III peroxidases (EC 1.11.1.7) consist of those peroxidases that are exclusive to plants [2]. In various previous studies, these peroxidases have been referred to by a multitude of abbreviations, including PRX, POX, POD, Px, and PER. In the present study, we will adopt the abbreviation “PRX” to represent class III peroxidases. These peroxidases (PRXs) are glycoproteins known for their thermal stability and can be found in the cell walls and vacuoles of plant tissues [3]. The structure of the PRX protein is characterized by highly conserved amino acid residues and consists of two components: a single peptide chain and a protoporphyrin IX domain [4]. Furthermore, two histidine residues engage with a heme group and eight cysteine residues within its internal framework to establish disulfide bridges, which are crucial for enzymatic catalytic activity [5].

Some studies have demonstrated that PRX proteins play crucial roles in various physiological processes in plants, such as lignin and liposome formation, cell wall protein cross-linking [6], auxin metabolism [7], cell growth, and elongation [8]. Furthermore, *PRX* genes can also enhance stress resistance by modulating the levels of reactive oxygen species (ROS) in plants [9]. For instance, the overexpression of the *OsPRX38* gene in *Arabidopsis* increased SOD, POD, and GST activities; lowered H_2_O_2_ levels; decreased electrolyte leakage and malondialdehyde contents; and thus improved arsenic stress tolerance [10]. Similarly, the overexpression of the *TaPRX-2A* gene in wheat enhanced SOD, POD, and CAT activities; lowered ROS levels; and improved salt and drought stress resistance [11,12]. The overexpression of the *IbPRX17* gene in sweet potatoes enhanced tolerance to salt and drought stresses by effectively scavenging ROS [13]. Furthermore, the overexpression of the *OsPRX30* gene in rice reduced the the bacterial blight resistance by reducing H_2_O_2_ contents [14]. Taken together, *PRX* genes are crucial for plant biological functions and responses to multiple biotic and abiotic stresses; therefore, a comprehensive analysis of these genes is essential to further explore their roles in plants.

*PRX* genes were first reported in Arabidopsis by Intapruk et al. [15]. Thereafter, numerous *PRX* genes have been extensively characterized across a variety of plant species due to the results of transcriptomic data. Currently, a total of 73 *PRX* genes have been identified in *Arabidopsis* [16]. Other significant discoveries included 138 *PRX* genes in rice [5], 124 *PRX* genes in soybean [17], and 102 *PRX* genes in potatoes [18]. Additional studies revealed the presence of 47 *PRX* genes in grapes [19], 82 *PRX* genes in sugarcane [20], and 75 *PRX* genes in carrot [21]. A comprehensive bioinformatics analysis of the *PRX* gene family will significantly enhance our understanding of their physiological functions and characteristics. Nevertheless, to date, there has been no genome-wide analysis performed on the *PRX* gene family in cucumber.

Cucumber is one of the most extensively cultivated vegetable crops globally and has important economic and social value. In 2022, the global cucumber planting area was more than 2.17 million hectares, resulting in a remarkable total production of approximately 94.72 million tons. In China, the cucumber planting area was approximately 1.31 million hectares, yielding more than 77.3 million tons, which constituted 60.37% of the world’s cucumber cultivation area and 81.61% of its total output [22]. Nevertheless, various adverse environmental factors, including extreme weather, low temperature, insufficient light, drought, salinity, and pollution from heavy metals, have severely hindered cucumber growth, resulting in a decline in yield and quality. Consequently, it is of significant importance to understand the mechanisms of stress response in cucumbers.

In this present study, we comprehensively investigated the *PRX* gene family in cucumbers using bioinformatics methods, including gene structure, chromosomal location, and phylogenetic relationship. Moreover, we examined the tissue-specific expression profiles of *CsPRX* genes and their expression patterns when subjected to various abiotic stresses, including treatments with NaCl, CdCl_2_, and PEG. Our findings will undoubtedly be helpful for in-depth research on the biological roles of *CsPRX* genes in cucumber.

## 2. Materials and Methods

### 2.1. Characterization of PRX Gene Family in Cucumber

The cucumber genome annotation files, coding sequences (CDS), and protein sequence files were obtained from the *Cucurbitaceae* database (CuCgenDB: the Index of /FTP/genome/cucumber/Chinese_long/v3, accessed on 11 April 2024). The reference sequences of the *PRX* gene family in *Arabidopsis* were obtained from the TAIR database (TAIR: https://www.arabidopsis.org/browse/genefamily/index.jsp, accessed on 11 April 2024) and preliminarily screened by Blast comparison. The HMM file of the conserved domain of the *PRX* gene family (PF00141) was obtained from the Pfam online website (https://www.ebi.ac.uk/interpro/, accessed on 11 April 2024), and then the hmmsearch program of the TBtools software v2.056 was used to search for the proteins containing the conserved domain in cucumber. NCBI CDD (CDD, http://www.ncbi.nlm.nih.gov/cdd, accessed on 12 April 2024) was used to verify the proteins. The physical and chemical properties of CsPRX proteins were conducted using ExPASy (https://web.expasy.org/protparam/, accessed on 12 April 2024). The subcellular localization of CsPRX proteins was predicted through the Wolfpsort online tool (https://wolfpsort.hgc.jp/, accessed on 13 April 2024).

### 2.2. Gene Structure and Phylogenetic Relationship of PRX Proteins

MEME (https://meme-suite.org/meme/, accessed on 13 April 2024) was utilized for the identification of conserved motifs in all the CsPRX proteins. TBtools was employed to display the conserved domain, along with the positions of introns and exons, as well as the chromosomal location of *CsPRX* genes. The phylogenetic tree of CsPRX proteins was generated through multiple sequence alignments, applying the Neighbor-Joining (NJ) method. For the syntenic analysis of *PRX* genes between *Arabidopsis* and cucumber, the One StepMC ScanX-Superfast program within TBtools was used. *C*is-regulatory elements (CREs) were predicted using the PlantCARE database (https://bioinformatics.psb.ugent.be/webtools/plantcare/html/, accessed on 13 April 2024).

### 2.3. Plant Materials and Treatments

In this study, a cucumber ‘Jinyou 1’ variety was utilized as plant material. The cucumber seeds were soaked in sterilized water at 55 °C for 15 min and then germinated at 28 ± 1 °C in sterile culture dishes with filter paper. The 5-day-old cucumber seedlings were transferred to 1/2 MS liquid medium with 100 mmol·L^−1^ NaCl solutions (salt stress), 125 mg·L^−1^ CdCl_2_ solutions (cadmium stress), and 10% PEG-6000 solutions (drought stress), respectively. Cucumber roots were harvested after 0, 6, 12, 24, and 48 h following stresses for RNA isolation.

### 2.4. Gene Expression Analysis

Transcriptome data for *CsPRX* genes in various tissues (PRJNA80167) were sourced from the *Cucurbitaceae* database. In this study, the read counts were characterized as fragments per kilobase of transcript per million fragments (FPKMs) for *CsPRX* genes. A heatmap of expression was generated using log_2_FPKM values. The expression patterns of four *CsPRX* genes subjected to NaCl, CdCl_2_, and PEG treatments were examined using real-time quantitative PCR (qRT-PCR) analysis. The total RNA of cucumber was isolated using the DNase I plant RNA extraction kit (Kangweishiji, Beijing, China, CW2598), and complementary DNA (cDNA) was synthesized using PrimeScript™ RTMaster Mix (Takara, Kusatsu, Japan, RR036A). The qRT-PCR analysis was conducted using TB Green^®^Premix Ex Taq™ Ⅱ (Takara, RR820A). The primer sequences for the four *CsPRX* genes and the reference gene (*CsActin*) are detailed in Supplementary Table S1. The relative expression levels of the four *CsPRX* genes were quantified employing the 2^−(∆∆Ct)^ method.

## 3. Results

### 3.1. Identification of PRX Genes in Cucumber

A total of 73 *PRX* candidate genes were identified by Blast and Pfam in cucumber, and 60 CsPRX proteins were obtained by CDD verification, which were renamed as CsPRX01-CsPRX60 according to their distribution on chromosomes. The number of amino acids (aas) encoded for the CsPRX proteins in cucumber ranged from 255 aa to 742 aa. The relative molecular weight (MW) was between 27.94 kD and 80.80 kD, and the theoretical isoelectric point (pI) was from 4.61 to 9.40. The Grand average of Hydropathicity (GRAVY) analysis showed that 6 proteins (CsPRX14, CsPRX27, CsPRX30, CsPRX32, CsPRX36, and CsPRX52) were hydrophobic, while the remaining 54 CsPRX proteins were hydrophilic. In addition, the instability index (II) of 29 CsPRX proteins was less than 40, ranging from 26.49 to 39.8, showing the stable characteristics. While, the II of 31 CsPRX proteins was greater than 40, ranging from 40.02 to 52.01, showing the unstable characteristics. Subcellular localization revealed that CsPRX proteins in cucumber were located in the chloroplasts (23), extracellular (14), vacuole membrane (10), endoplasmic reticulum (5), mitochondria (3), cytoplasm (2), plasma membrane (2), and cell membrane (1). Detailed information on the CsPRX proteins in cucumber is shown in Table 1.

### 3.2. Chromosomal Location of CsPRX Genes

**Figure 1:**
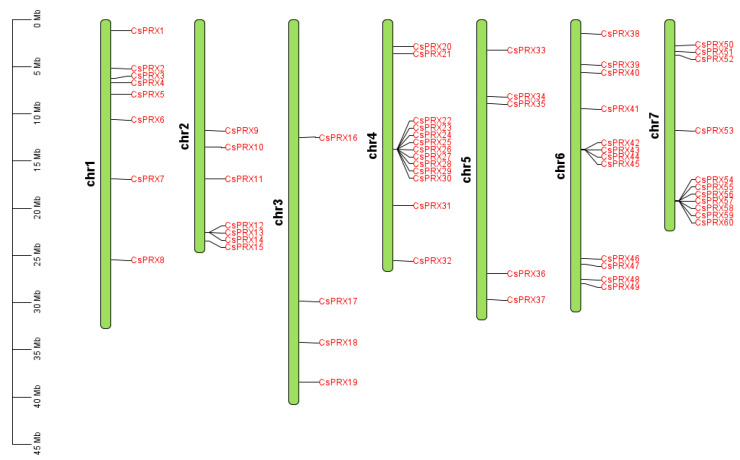
Chromosomal location of *CsPRX* genes in cucumber.

To reveal the distribution of *CsPRX* genes across different chromosomes in cucumber, a chromosomal graph was constructed. A total of 60 members of the *CsPRX* family were located on seven distinct chromosomes (Figure 1). Specifically, chromosome 1 housed 8 *CsPRX* genes, chromosome 2 contained 7 *CsPRX* genes, chromosome 3 had 4 *CsPRX* genes, chromosome 4 comprised 13 *CsPRX* genes, chromosome 5 included 5 *CsPRX* genes, chromosome 6 featured 12 *CsPRX* genes, and chromosome 7 held 11 *CsPRX* genes.

### 3.3. Phylogenetic Analysis of CsPRX Genes

**Figure 2:**
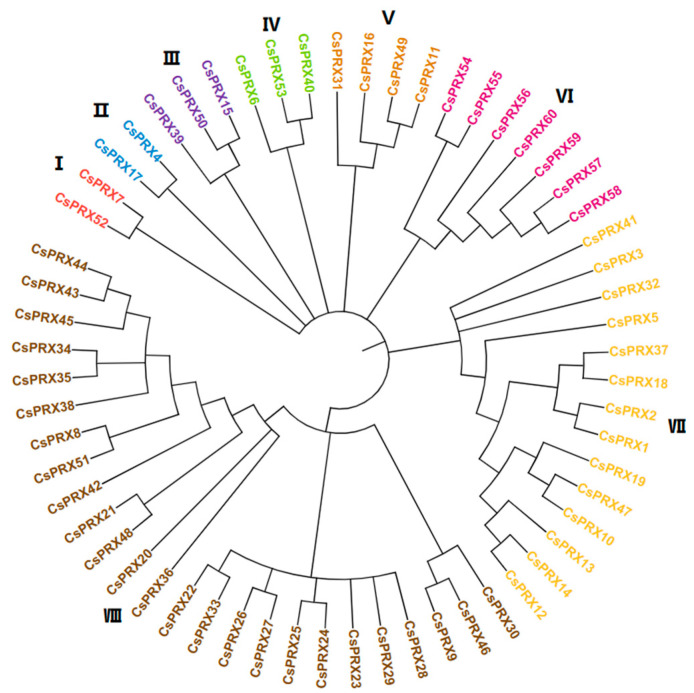
Phylogenetic tree of Cs*PRX* genes in cucumber. I, II, III, IV, V, VI, VII, VIII indicated different groups of Cs*PRX* genes.

**Figure 3:**
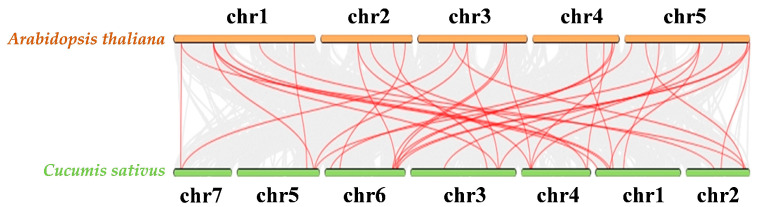
Synteny relationships of the *PRX* gene family in cucumber and *Arabidopsis*. Identified collinear genes highlighted as red lines.

To elucidate the evolutionary relationships within the *CsPRX* gene family, a phylogenetic tree was generated utilizing the Neighbor-Joining (NJ) method (Figure 2). The findings revealed that 60 *CsPRX* genes were divided into 8 groups: groups I and II each contained 2 *CsPRX* genes, groups III and IV included 3 *CsPRX* genes, group V had 4 *CsPRX* genes, group VI contained 7 *CsPRX* genes, group VII included 14 *CsPRX* genes, and group VIII had 25 *CsPRX* genes. Furthermore, the syntenic relationships among the *PRX* family genes from cucumber and *Arabidopsis* were examined to further understand the evolution of *PRX* genes (Figure 3). The results indicated that a total of 36 syntenic gene pairs of *PRX* genes were identified between cucumber and *Arabidopsis*, suggesting a high level of conservation among the *PRX* family members throughout evolution.

### 3.4. Conserved Motifs and Gene Structures of CsPRX Genes

**Figure 4:**
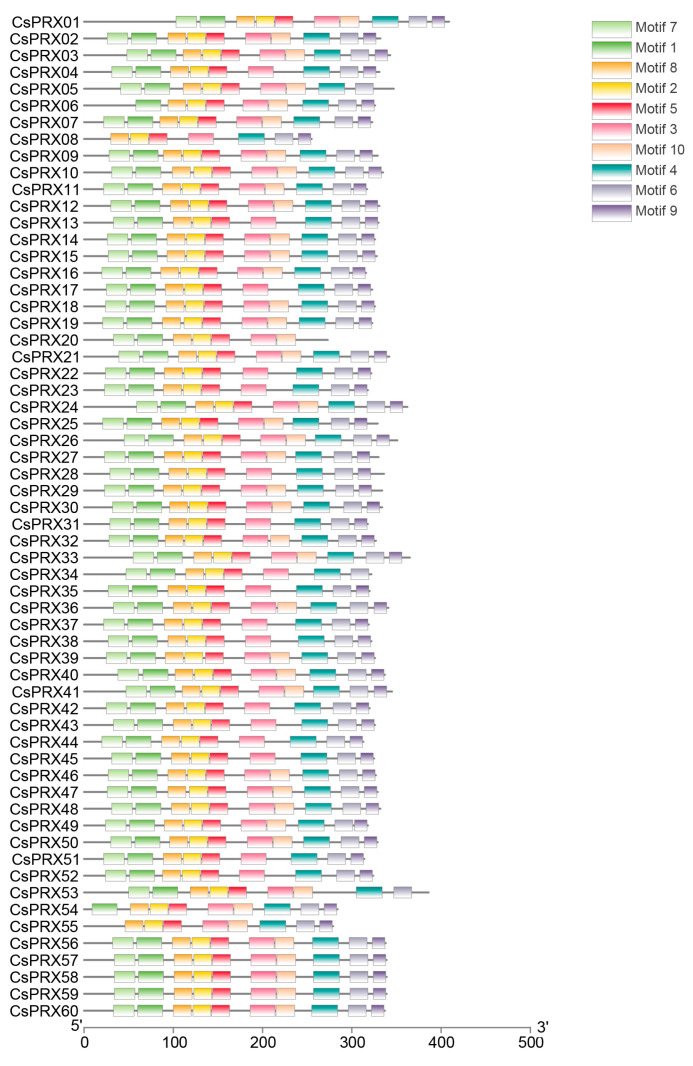
Conserved motifs of CsPRX proteins in cucumber.

**Figure 5:**
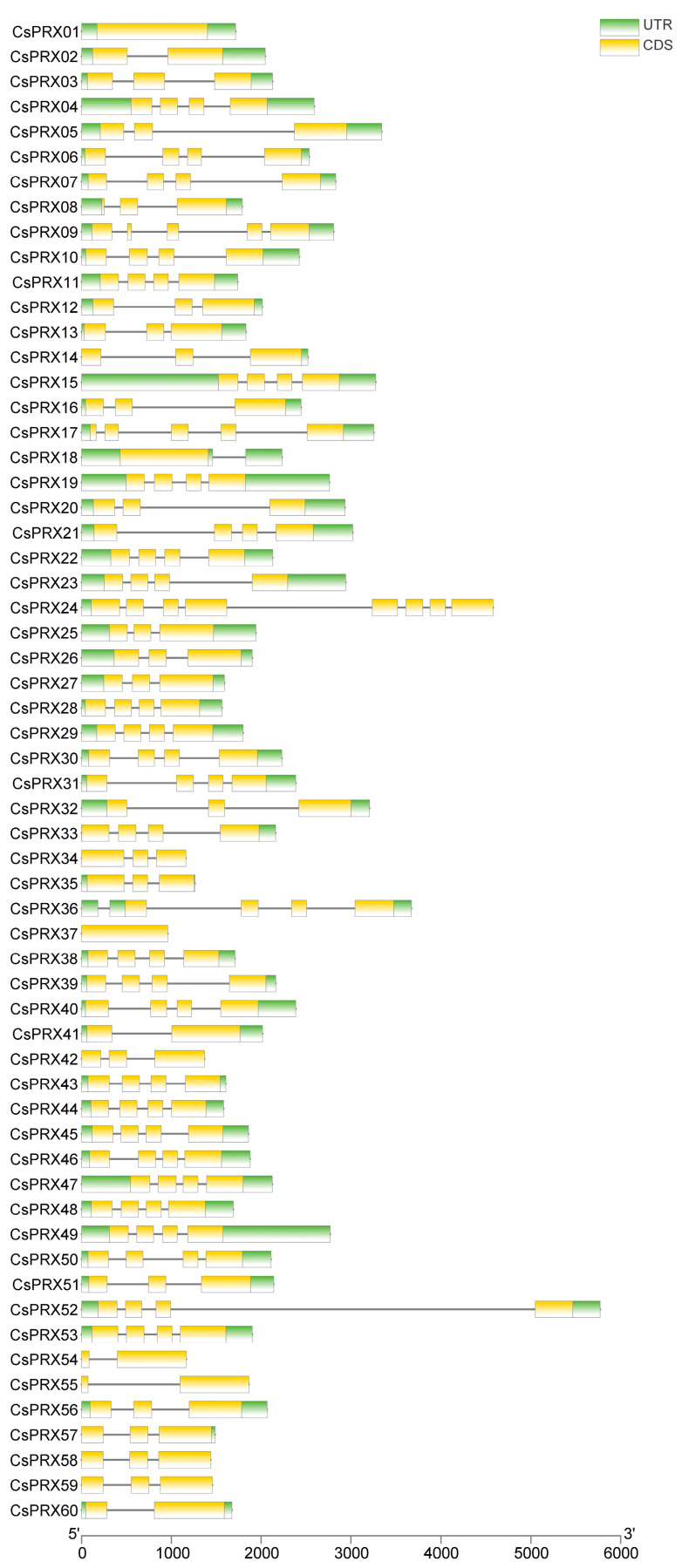
Gene structure of *CsPRX* genes in cucumber.

MEME was utilized to identify the conserved motifs of the CsPRX proteins in cucumber, resulting in the identification of 10 divergent motifs (Figure 4). Among the 60 members of the CsPRX family, 37 CsPRX proteins contained 10 motifs, 19 proteins had 9 motifs, and 4 proteins included 7–8 motifs. Notably, among the ten motifs, motifs 8, motif 2, motif 5, and motif 3 were present in all 60 CsPRX protein sequences and appeared in the same order, indicating their conservation. The gene structure analysis of the *CsPRX* genes revealed that the number of introns varied from 0 to 7, with most genes containing between 1 and 4 introns. *CsPRX24* had the highest number of introns (7), while both *CsPRX1* and *CsPRX37* lacked introns (Figure 5). Furthermore, the structural analysis of the *CsPRX* family genes indicated that most *CsPRX* genes contained both 5’ UTR and 3’ UTR; however, *CsPRX24* contained only a 5’ UTR, while *CsPRX14*, *CsPRX33*, and *CsPRX57* contained only a 3′ UTR (Figure 5).

### 3.5. Cis-Regulatory Elements of CsPRX Genes in Cucumber

**Figure 6:**
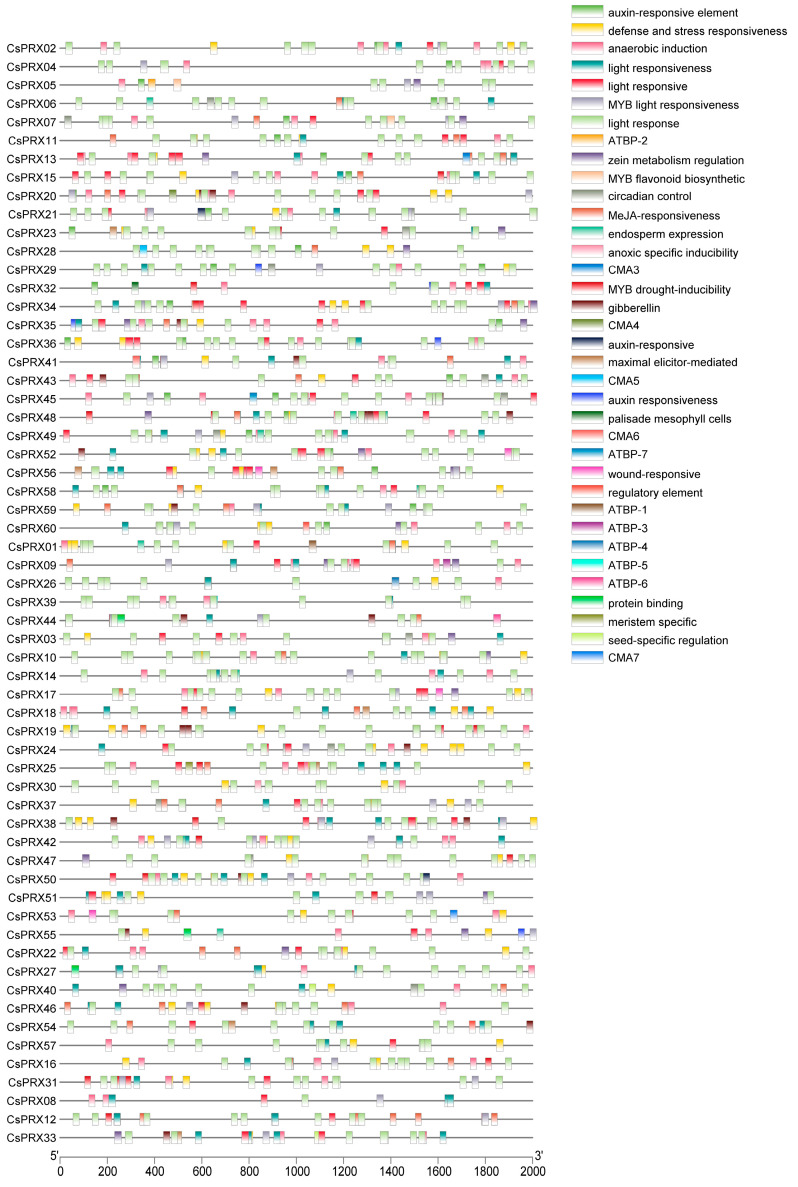
CREs of *CsPRX* genes in cucumber.

Transcription factors have crucial roles in the regulation of gene expression by binding to CREs located within the promoter regions of genes. To investigate the CREs of the CsPRX family genes in cucumber, we scanned the 2000 bp promoter regions of CsPRX genes by utilizing the online PlantCARE database. As illustrated in Figure 6, 175 CREs were categorized into light response elements, plant hormone response elements, stress response elements, and growth and development elements, according to their putative functions. The light response elements included the CTT-motif, chs-CMA1a, GATA-motif, L-box, 3-AF1 binding site, chs-CMA2b, G-box, GT1-motif, GCN4-motif, chs-CMA2a, CAG-motif, LAMP-element, MRE, and TCC-motif, among others. The plant hormone response elements comprised ABRE, MBSI, CGTCA-motif, TGACG-motif, TATC-box, P-box, and ARE-motif. The stress response elements included TC-rich repeats, STRE, MBS, and LTR. The growth and development elements encompassed O2-site, GCN4-motif, TGA-element, AuxRE, and TGA-box. Additionally, a substantial number of promoter/enhancer elements like the TATA-box and CAAT-box, as well as binding elements like the W-box, MYB recognition site, and MYC, were also identified. Thus, the variety of CREs present within the CsPRX genes suggested their varied roles in biological processes, including responses to light, plant hormones, and environmental stress.

### 3.6. Tissue Specificity of CsPRX Genes in Cucumber

**Figure 7:**
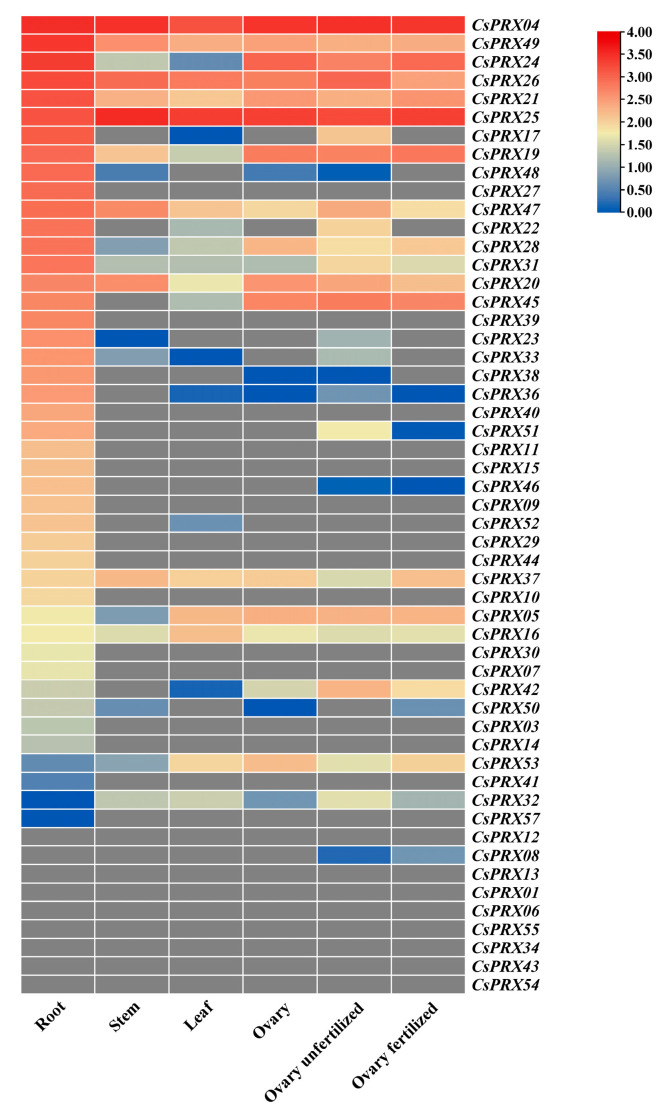
Transcriptional levels of the *CsPRX* genes in the different tissues.

To further investigate the tissue specificity of the *CsPRX* genes, transcript data from various cucumber tissues were sourced from an accessible genome database. The tissues examined included the root, stem, leaf, ovary, unfertilized ovary, and fertilized ovary. Based on the FPKM values, the *CsPRX* genes exhibited varying levels of expression across these six tissues, with *CsPRX04*, *CsPRX49*, *CsPRX26*, *CsPRX21*, and *CsPRX25* generally showing higher expression levels in all the tissues. Furthermore, we observed that the majority of the *CsPRX* genes displayed higher expression in the root compared to the other tissues. The expression abundances of *CsPRX25* and *CsPRX37* were significantly greater in the stem than in the other three tissues. *CsPRX16* demonstrated relatively high expression in the leaf. Additionally, *CsPRX05*, *CsPRX45*, and *CsPRX53* exhibited higher expression levels in the ovary, unfertilized ovary, and fertilized ovary than in the other three tissues (Figure 7). These results suggested that the expression of *CsPRX* genes was regulated in a tissue-specific manner within cucumber, and the unique expression patterns observed across the different tissues reflected their various functions in cucumber.

### 3.7. Expression Profiles of CsPRX Genes under Abiotic Stresses

**Figure 8:**
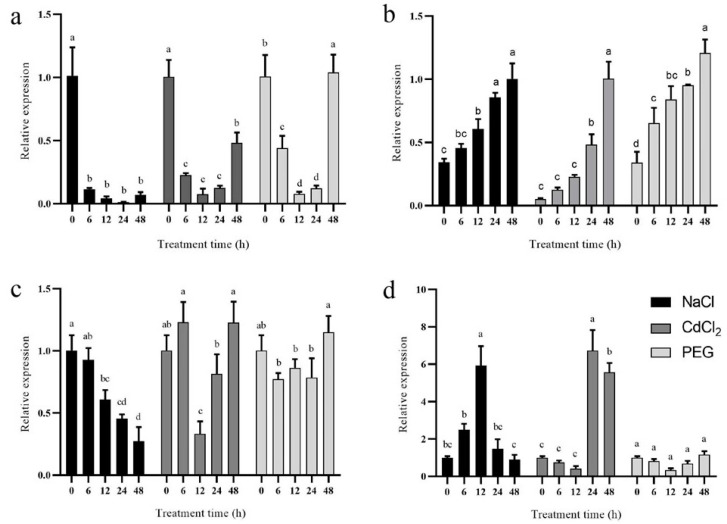
Expression levels of *CsPRX* genes in response to NaCl, CdCl_2_, and PEG stresses: (**a**) *CsPRX04*; (**b**) *CsPRX17*; (**c**) *CsPRX30*; (**d**) *CsPRX36*. Different lower-case letter indicate significant differences in means (*p* < 0.05).

*CsPRX* genes are not only involved in plant developmental processes but also in response to multiple abiotic stresses. The CRE analysis of promoter sequences discovered the presence of several stress response elements within the *CsPRX* genes. To further explore how the *CsPRX* genes respond to NaCl stress, CdCl_2_ stress, and PEG stress, qRT-PCR experiments were conducted to measure the relative expression levels. The expression profiles of these four genes (*CsPRX04*, *CsPRX17*, *CsPRX30*, and *CsPRX36*) demonstrated considerable variability across the various treatments (Figure 8). Specifically, the expression levels of *CsPRX04* and *CsPRX30* were down-regulated in response to NaCl stress. Conversely, *CsPRX17* was significantly induced by NaCl stress, exhibiting higher expression levels from 12 h to 24 h after treatment. Unlike the other three genes, *CsPRX36* displayed an initial increase followed by a decrease during NaCl stress. Additionally, the expression level of *CsPRX04* was suppressed by CdCl_2_ stress. The transcript levels of *CsPRX17* and *CsPRX36* were remarkably induced at both 24 h and 48 h of CdCl_2_ stress. *CsPRX30* was induced after 6 h of CdCl_2_ stress, followed by a subsequent decrease and then a rise. Under PEG stress, the expression level of *CsPRX04* showed a significant decrease initially, followed by an increase, while *CsPRX17* exhibited a notable upward trend. *CsPRX30* and *CsPRX36*, however, were not significantly affected by PEG stress. Collectively, these observations implied functional divergence among the *CsPRX* genes in their response to abiotic stresses. The *CsPRX17* gene was significantly induced by NaCl, CdCl_2_, and PEG stresses, exhibiting higher expression levels from 12 h to 24 h after treatment, suggesting a vital role of the *CsPRX17* gene in response to environmental stresses.

## 4. Discussion

Peroxidases are enzymes found specifically in plants that are crucial for multiple stress responses during plant development. Although extensive studies have been carried out on the *PRX* gene family across various plant species, there is still a deficiency in research concerning the *CsPRX* genes in cucumber. In this research, we identified 60 *CsPRX* genes in cucumber, designated *CsPRX1-60* (Table 1). This number is slightly less than that found in *Arabidopsis* (73) and carrot (75), but greater than that in grape (47). Furthermore, our analysis revealed that motifs 8, 2, 5, and, 3 were present in all 60 CsPRX protein sequences in the same order, indicating a level of conservation among CsPRX proteins. It is known that variations in the intron/exon structures contribute to gene diversity, driving the evolution of multi-gene families and resulting in diverse functional outcomes in gene evolution [18]. In this study, we observed considerable variability in the number of introns among *CsPRX* genes, ranging from 0 to 7. Of the 60 *CsPRX* genes, 29 genes contained three introns and four exons. This pattern was also noted as a significant proportion in *Arabidopsis* [16], rice [5], and potato [18], suggesting that this represented an ancestral intronic model for *PRX* genes. In addition, the syntenic analysis of *PRX* genes between *Arabidopsis* and cucumber also implied the high level of conservation among the *PRX* family members throughout evolution.

The patterns of gene expression and the presence of CREs in gene family members provide significant insights into gene functions and regulatory mechanisms [20]. It was reported that the *ShPRX* family genes were involved in regulating plant hormones, responding to abiotic stresses, managing tissue-specific cell cycles, and controlling circadian rhythms through the analysis of CREs. In this study, the CREs of *CsPRX* genes were classified into four groups, suggesting that these genes served as regulators actively participating in cucumber development, and multiple stress responses.

The expression patterns of *CsPRX* genes were examined, revealing that these genes were present in various tissues, such as the root, stem, leaf, ovary, unfertilized ovary, and fertilized ovary. This indicated that *CsPRX* genes exhibited extensive expression in cucumber, although variations in expression patterns were observed. In the case of sugarcane, of the 44 *ShPRX* genes with high expression levels, 26 and 43 were detected in the leaf and stem, respectively, suggesting that most of these genes might play significant roles in these tissues [20]. In the current study, the expression profiles of *CsPRX* genes demonstrated variation across various tissues. For instance, *CsPRX25* and *CsPRX37* were significantly higher in the stem, while *CsPRX16* exhibited relatively higher expression in the leaf. Additionally, *CsPRX05*, *CsPRX45*, and *CsPRX53* showed elevated expression levels in the ovary. These findings suggested the unique functions of *CsPRX* genes in cucumber development. Notably, a majority of *CsPRX* genes exhibiting higher expression levels were identified in the root of cucumber. Similar observations have been documented in *Arabidopsis* [16], rice [5], maize [23], and potato [18]. For example, the overexpression of *AtPRX01*, *AtPRX44*, and *AtPRX73* has been shown to promote root hair elongation in *Arabidopsis* [24]. Furthermore, a decrease in the expression of *PRX2/ATPRX1*, *PRX8*, *PRX35*, and *PRX73* negatively affected cell elongation, vegetative growth, and the development of vascular structures in *Arabidopsis* [25]. These findings provide basic information about the role of *CsPRX* genes in cucumber root functionality, and the gene expression patterns and functions need further validation.

Abiotic stresses significantly hinder plant growth and productivity, leading to economic losses. Previous studies have reported that *PRX* genes can be markedly influenced by abiotic stresses, exhibiting distinct expression patterns in soybean [17], grapevine [19], and potato [18]. Plant peroxidase is involved in various cell growth and development processes, responding to both abiotic and biotic stresses through plant hormone signaling pathways [26]. Numerous researchers have indicated that plants produce ROS under stresses from salt and Cd, with the *PRX* family genes playing crucial roles in mitigating these stresses by scavenging ROS levels [12,27,28]. In wheat, the overexpression of the *TaPRX-2A* gene has been shown to trigger the abscisic acid (ABA) signaling pathway and enhance antioxidant enzyme activity, leading to a decrease in ROS levels and an increase in osmotic metabolites, thereby improving salt tolerance [12]. Furthermore, in sugarcane, *ShPRX* genes enhanced tolerance to Cd and salt stresses by activating the antioxidant defense mechanism and reducing ROS levels [20]. Previous researches has also indicated that drought stress enhanced the POD activity [29], and higher expression levels of *POD* genes have been shown to be associated with enhanced tolerance to drought and osmotic stresses in plants [30,31]. The *GsPOD40* gene promoted drought resistance in soybean by modulating critical physiological and biochemical pathways involved in defense responses [17]. To verify the response of *CsPRX* genes under various environmental stimuli and cucumber development, the qRT-PCR analysis of four *CsPRX* genes in response to NaCl, CdCl_2_, and PEG stresses demonstrated a range of differential expression, and the *CsPRX17* gene was significantly induced by NaCl, CdCl_2_, and PEG stresses, suggesting a vital role of the *CsPRX17* gene in response to environmental stresses. Further studies are needed to elucidate the functions of the *CsPRX17* gene through either gain-of-function or loss-of-function experiments in cucumber.

## 5. Conclusions

In this study, 60 *CsPRX* genes were identified in cucumber by comprehensive investigation. Motif 8, motif 2, motif 5, and motif 3 consistently appeared across all 60 CsPRX protein sequences, indicating the conservation of CsPRX proteins. Furthermore, we analyzed the expression patterns of the *CsPRX* genes across various tissues and their responses to abiotic stresses in cucumber, indicating that a majority of the *CsPRX* genes might be essential for root development, and the *CsPRX17* gene might be linked to environmental stresses. Our results provided a systematic insight into the characterization of *CsPRX* genes and highlighted the possible functions of the *CsPRX17* gene in cucumber development and stress responses. This work lays the foundation for future investigations into the roles and regulatory mechanisms of *CsPRX* genes in particular environments.

## Figures and Tables

**Table 1:** Physical and chemical properties of CsPRX proteins in cucumber.

Name	Gene ID	AA	MW (Da)	pI	II	GRAVY	Subcellular Localization
*CsPRX1*	*CsaV3_1G001790.1*	409	44,782.37	6.57	36.19	−0.106	endoplasmic reticulum
*CsPRX2*	*CsaV3_1G008260.1*	332	36,902.27	6.36	30.43	−0.096	extracellular
*CsPRX3*	*CsaV3_1G009980.1*	343	38,125.62	5.84	40.75	−0.173	extracellular
*CsPRX4*	*CsaV3_1G010780.1*	331	37,686.25	8.47	41.32	−0.352	vacuole membrane
*CsPRX5*	*CsaV3_1G012650.1*	347	37,950.81	9.30	39.59	−0.224	chloroplasts
*CsPRX6*	*CsaV3_1G014970.1*	255	35,741.98	6.53	40.29	−0.029	chloroplasts
*CsPRX7*	*CsaV3_1G030170.1*	323	35,722.78	5.63	32.33	−0..155	extracellular
*CsPRX8*	*CsaV3_1G040180.1*	255	27,942.32	5.15	35.64	−0.314	cytoplasm
*CsPRX9*	*CsaV3_2G014160.1*	329	36,569.78	4.96	30.07	−0.047	extracellular
*CsPRX10*	*CsaV3_2G016300.1*	335	36,566.83	8.86	34.73	−0.092	vacuole membrane
*CsPRX11*	*CsaV3_2G024340.1*	317	34,683.64	9.09	49.80	−0.095	extracellular
*CsPRX12*	*CsaV3_2G034090.1*	331	35,690.26	4.74	36.49	−0.001	vacuole membrane
*CsPRX13*	*CsaV3_2G034100.1*	330	35,735.71	8.74	37.72	−0.137	cytoplasm
*CsPRX14*	*CsaV3_2G034110.1*	326	34,863.86	6.93	26.49	0.080	endoplasmic reticulum
*CsPRX15*	*CsaV3_2G035150.1*	328	36,082.20	8.10	40.23	−0.139	vacuole membrane
*CsPRX16*	*CsaV3_3G016670.1*	316	34,565.47	7.56	38.95	−0.053	cell membrane
*CsPRX17*	*CsaV3_3G035690.1*	323	36,388.92	8.31	34.08	−0.181	chloroplasts
*CsPRX18*	*CsaV3_3G042040.1*	326	35,890.94	8.63	34.12	−0.196	chloroplasts
*CsPRX19*	*CsaV3_3G047080.1*	323	36,036.27	9.06	27.15	−0.170	extracellular
*CsPRX20*	*CsaV3_4G004610.1*	273	30,455.90	5.40	41.04	−0.041	chloroplasts
*CsPRX21*	*CsaV3_4G005430.1*	342	37,447.46	9.18	49.20	−0.294	extracellular
*CsPRX22*	*CsaV3_4G023590.1*	322	34,297.21	4.94	42.45	−0.153	vacuole membrane
*CsPRX23*	*CsaV3_4G023610.1*	318	34,844.31	6.43	52.01	−0.196	vacuole membrane
*CsPRX24*	*CsaV3_4G023620.1*	742	80,803.84	6.17	40.02	−0.229	plasma membrane
*CsPRX25*	*CsaV3_4G023630.1*	329	36,469.33	6.24	46.57	−0.264	extracellular
*CsPRX26*	*CsaV3_4G023640.1*	351	38,281.28	9.11	43.07	−0.006	chloroplasts
*CsPRX27*	*CsaV3_4G023650.1*	330	35,502.55	5.51	37.98	0.102	extracellular
*CsPRX28*	*CsaV3_4G023660.1*	336	36,453.37	8.34	41.41	−0.224	chloroplasts
*CsPRX29*	*CsaV3_4G023670.1*	334	35,554.70	4.61	43.64	−0.082	vacuole membrane
*CsPRX30*	*CsaV3_4G023680.1*	334	36,476.81	5.63	42.78	0.043	vacuole membrane
*CsPRX31*	*CsaV3_4G029960.1*	318	35,080.10	8.10	44.63	−0.092	chloroplasts
*CsPRX32*	*CsaV3_4G036360.1*	327	34,873.18	5.10	37.76	0.006	plasma membrane
*CsPRX33*	*CsaV3_5G005000.1*	365	39,538.89	5.16	42.23	−0.038	chloroplasts
*CsPRX34*	*CsaV3_5G012680.1*	322	35,243.14	8.16	38.21	−0.012	endoplasmic reticulum
*CsPRX35*	*CsaV3_5G012840.1*	320	34,594.32	7.57	43.66	−0.099	extracellular
*CsPRX36*	*CsaV3_5G033660.1*	341	37,746.19	5.24	30.42	0.024	extracellular
*CsPRX37*	*CsaV3_5G037450.1*	319	35,013.07	8.36	42.21	−0.002	chloroplasts
*CsPRX38*	*CsaV3_6G002170.1*	322	34,128.24	8.63	48.52	−0.097	chloroplasts
*CsPRX39*	*CsaV3_6G005630.1*	326	36,159.26	9.28	49.78	−0.187	chloroplasts
*CsPRX40*	*CsaV3_6G006890.1*	337	37,933.75	9.14	47.17	−0.461	vacuole membrane
*CsPRX41*	*CsaV3_6G013360.1*	345	38,494.56	9.34	33.68	−0.045	vacuole membrane
*CsPRX42*	*CsaV3_6G018990.1*	320	34,795.30	7.58	27.51	−0.160	chloroplasts
*CsPRX43*	*CsaV3_6G019010.1*	326	35,456.53	9.26	40.02	−0.196	extracellular
*CsPRX44*	*CsaV3_6G019020.1*	313	34,396.09	6.99	32.03	−0.236	chloroplasts
*CsPRX45*	*CsaV3_6G019040.1*	325	35,404.37	9.12	42.38	−0.123	chloroplasts
*CsPRX46*	*CsaV3_6G043090.1*	327	36,656.70	6.43	33.14	−0.165	extracellular
*CsPRX47*	*CsaV3_6G043930.1*	329	36,083.28	9.36	36.70	−0.077	chloroplasts
*CsPRX48*	*CsaV3_6G046710.1*	332	36,387.16	9.04	46.80	−0.319	chloroplasts
*CsPRX49*	*CsaV3_6G047430.1*	318	34,499.38	9.06	39.26	−0.084	endoplasmic reticulum
*CsPRX50*	*CsaV3_7G003750.1*	329	35,669.05	9.20	36.63	−0.030	chloroplasts
*CsPRX51*	*CsaV3_7G005720.1*	314	34,009.52	8.60	29.85	−0.181	chloroplasts
*CsPRX52*	*CsaV3_7G006200.1*	324	35,012.99	6.11	41.44	0.013	chloroplasts
*CsPRX53*	*CsaV3_7G022880.1*	386	43,158.60	5.79	41.10	−0.294	chloroplasts
*CsPRX54*	*CsaV3_7G030360.1*	284	30,755.02	6.83	36.75	−0.134	extracellular
*CsPRX55*	*CsaV3_7G030370.1*	279	30,498.63	5.20	38.27	−0.200	endoplasmic reticulum
*CsPRX56*	*CsaV3_7G030380.1*	338	36,567.71	9.40	42.64	−0.148	chloroplasts
*CsPRX57*	*CsaV3_7G030390.1*	339	36,860.73	8.04	41.24	−0.180	mitochondria
*CsPRX58*	*CsaV3_7G030400.1*	339	36,852.71	8.32	39.80	−0.183	mitochondria
*CsPRX59*	*CsaV3_7G030410.1*	339	36,896.89	8.03	42.37	−0.158	chloroplasts
*CsPRX60*	*CsaV3_7G030420.1*	337	36,908.94	8.62	43.11	−0.236	mitochondria

## Data Availability

The data are available upon request from the corresponding author.

## Supplementary Materials

The following supporting information can be downloaded at: https://www.mdpi.com/article/10.3390/genes15101245/s1, Table S1: Primer sequences for *CsPRX* genes.

## References

[1] Hiraga S., Ichinose C., Onogi T., Niki H., Yamazoe M. (2000). Bidirectional migration of SeqA-bound hemimethylated DNA clusters and pairing of *oriC* copies in *Escherichia coli*. Genes Cells.

[2] Hiraga S., Sasaki K., Ito H., Ohashi Y., Matsui H. (2001). A large family of class III plant peroxidases. Plant Cell Physiol..

[3] Passardi F., Cosio C., Penel C., Dunand C. (2005). Peroxidases have more functions than a Swiss army knife. Plant Cell Rep..

[4] Welinder K.G., Penel C., Gaspar T., Greppin H. (1992). Plant peroxidases: Structure-function relationships. Plant Peroxidases.

[5] Passardi F., Longet D., Penel C., Dunand C. (2004). The class III peroxidase multigenic family in rice and its evolution in land plants. Phytochemistry.

[6] Barceló A.R., Pomar F. (2001). Oxidation of cinnamyl alcohols and aldehydes by a basic peroxidase from lignifying Zinnia elegans hypocotyls. Phytochemistry.

[7] Hiraga S., Yamamoto K., Ito H., Sasaki K., Matsui H., Honma M., Nagamura Y., Sasaki T., Ohashi Y. (2000). Diverse expression profiles of 21 rice peroxidase genes. FEBS Lett..

[8] Schopfer P., Liszkay A., Bechtold M.K., Frahry G., Wagner A. (2002). Evidence that hydroxyl radicals mediate auxin-induced extension growth. Planta.

[9] Almagro L., Gómez Ros L.V., Belchi-Navarro S., Bru R., Ros Barceló A., Pedreño M.A. (2009). Class III peroxidases in plant defence reactions. J. Exp. Bot..

[10] Kidwai M., Dhar Y.V., Gautam N.K., Tiwari M., Ahmad I.Z., Asif M.H., Chakrabarty D. (2019). Oryza sativa class III peroxidase (*OsPRX38*) overexpression in *Arabidopsis thaliana* reduces arsenic accumulation due to apoplastic lignification. J. Hazard. Mater..

[11] Su P., Sui C., Niu Y., Li J., Wang S., Sun F., Yan J., Guo S. (2023). Comparative transcriptomic analysis and functional characterization reveals that the class III peroxidase gene TaPRX-2A regulates drought stress tolerance in transgenic wheat. Front. Plant Sci..

[12] Su P., Yan J., Li W., Wang L., Wang L., Zhao J., Ma X., Li A., Wang H., Kong L. (2020). A member of wheat class III peroxidase gene family, TaPRX-2A, enhanced the tolerance of salt stress. BMC Plant Biol..

[13] Zhang H., Wang Z., Li X., Gao X., Dai Z., Cui Y., Zhi Y., Liu Q., Zhai H., Gao S. (2022). The IbBBX24-IbTOE3-IbPRX17 module enhances abiotic stress tolerance by scavenging reactive oxygen species in sweet potato. New Phytol..

[14] Liu H., Dong S., Li M., Gu F., Yang G., Guo T., Chen Z., Wang J. (2021). The class III peroxidase gene OsPrx30, transcriptionally modulated by the AT-hook protein OsATH1, mediates rice bacterial blight-induced ROS accumulation. J. Integr. Plant Biol..

[15] Intapruk C., Higashimura N., Yamamoto K., Okada N., Shinmyo A., Takano M. (1991). Nucleotide sequences of two genomic DNAs encoding peroxidase of *Arabidopsis thaliana*. Gene.

[16] Tognolli M., Penel C., Greppin H., Simon P. (2002). Analysis and expression of the class III peroxidase large gene family in *Arabidopsis thaliana*. Gene.

[17] Aleem M., Riaz A., Raza Q., Aleem M., Aslam M., Kong K., Atif R.M., Kashif M., Bhat J.A., Zhao T. (2022). Genome-wide characterization and functional analysis of class III peroxidase gene family in soybean reveal regulatory roles of GsPOD40 in drought tolerance. Genomics.

[18] Yang X., Yuan J., Luo W., Qin M., Yang J., Wu W., Xie X. (2020). Genome-wide identification and expression analysis of the class III peroxidase gene family in potato (*Solanum tuberosum* L.). Front. Genet..

[19] Xiao H., Wang C., Khan N., Chen M., Fu W., Guan L., Leng X. (2020). Genome-wide identification of the class III POD gene family and their expression profiling in grapevine (*Vitis vinifera* L.). BMC Genom..

[20] Shang H., Fang L., Qin L., Jiang H., Duan Z., Zhang H., Yang Z., Cheng G., Bao Y., Xu J. (2023). Genome-wide identification of the class III peroxidase gene family of sugarcane and its expression profiles under stresses. Front. Plant Sci..

[21] Meng G., Fan W., Rasmussen S.K., Ppb B. (2021). Characterisation of the class III peroxidase gene family in carrot taproots and its role in anthocyanin and lignin accumulation. Plant Physiol. Biochem..

[22] Food and Agriculture Organization of the United Nations (2023). FAO-STAT Statistical Database.

[23] Wang Y., Wang Q., Zhao Y., Han G., Zhu S. (2015). Systematic analysis of maize class III peroxidase gene family reveals a conserved subfamily involved in abiotic stress response. Gene.

[24] Marzol E., Borassi C., Carignani Sardoy M., Ranocha P., Aptekmann A.A., Bringas M., Pennington J., Paez-Valencia J., Martínez Pacheco J., Rodríguez-Garcia D.R. (2022). Class III peroxidases PRX01, PRX44, and PRX73 control root hair growth in *Arabidopsis thaliana*. Int. J. Mol. Sci..

[25] Jeong Y.J., Kim Y.C., Lee J.S., Kim D.G., Lee J.H. (2022). Reduced expression of PRX2/ATPRX1, PRX8, PRX35, and PRX73 affects cell elongation, vegetative growth, and vasculature structures in *Arabidopsis thaliana*. Plants.

[26] Xue Y.J., Tao L., Yang Z.M. (2008). Aluminum-induced cell wall peroxidase activity and lignin synthesis are differentially regulated by jasmonate and nitric oxide. J. Agric. Food Chem..

[27] Chiang H.C., Lo J.C., Yeh K.C. (2006). Genes associated with heavy metal tolerance and accumulation in Zn/Cd hyperaccumulator *Arabidopsis* halleri: A genomic survey with cDNA microarray. Environ. Sci. Technol..

[28] Kidwai M., Ahmad I.Z., Chakrabarty D. (2020). Class III peroxidase: An indispensable enzyme for biotic/abiotic stress tolerance and a potent candidate for crop improvement. Plant Cell Rep..

[29] Hu W., Huang C., Deng X., Zhou S., Chen L., Li Y., Wang C., Ma Z., Yuan Q., Wang Y. (2013). TaASR1, a transcription factor gene in wheat, confers drought stress tolerance in transgenic tobacco. Plant Cell Environ..

[30] Choi H.W., Hwang B.K. (2012). The pepper extracellular peroxidase CaPO2 is required for salt, drought and oxidative stress tolerance as well as resistance to fungal pathogens. Planta.

[31] Kumar S., Jaggi M., Sinha A.K. (2012). Ectopic overexpression of vacuolar and apoplastic *Catharanthus roseus* peroxidases confers differential tolerance to salt and dehydration stress in transgenic tobacco. Protoplasma.

