# Supplementary material for: Molecular Characterization of Peroxidase (PRX) Gene Family in Cucumber
Source: Genes (Basel). 2024 Sep 25;15(10):1245. doi: 10.3390/genes15101245 (PMC11507654; doi:10.3390/genes15101245)
Supplement: Supplementary file 1 [file genes-15-01245-s001.zip › genes-3181048-supplementary.pdf]

Table S1 Primer sequences for *CsPRX* genes

Primer name	Primer Sequence (5'-3')
Actin-F	CAACCATAAACGATGCCGA
Actin-R	AGCCTTGCGACCATACTCC
CsPRX04-F	TGGGTATTGACACCTCTGGAGTGG
CsPRX04-R	AGGGTTCAGCACAGGGTCTACTTC
CsPRX17-F	TTGTGCTGACATCGTTGCTCTC
CsPRX17-R	ATGCGTTGTGCTGTGGAACC
CsPRX30-F	ACGATATGCGAATGGCTGCTTCC
CsPRX30-R	TCCCCATCACTCCCATCCAACAG
CsPRX36-F	AGGGTCTCTCGGTTACTGACATGG
CsPRX36-R	CGGGTTGTTTGGATCTGAAGTTGC
